# Association Mechanism of Peptide-Coated Metal Nanoparticles with Model Membranes: A Coarse-Grained Study

**DOI:** 10.1021/acs.jctc.1c00127

**Published:** 2021-06-02

**Authors:** Sebastian Franco-Ulloa, Daniela Guarnieri, Laura Riccardi, Pier Paolo Pompa, Marco De Vivo

**Affiliations:** † Molecular Modeling and Drug Discovery Lab, Istituto Italiano di Tecnologia, Via Morego 30, 16163 Genova, Italy; ‡ Dipartimento di Chimica e Biologia “A. Zambelli”, Università degli Studi di Salerno, Via Giovanni Paolo II, 132, Fisciano, l-84084 Salerno, Italy; § Nanobiointeractions & Nanodiagnostics, Istituto Italiano di Tecnologia, Via Morego 30, 16163 Genova, Italy

## Abstract

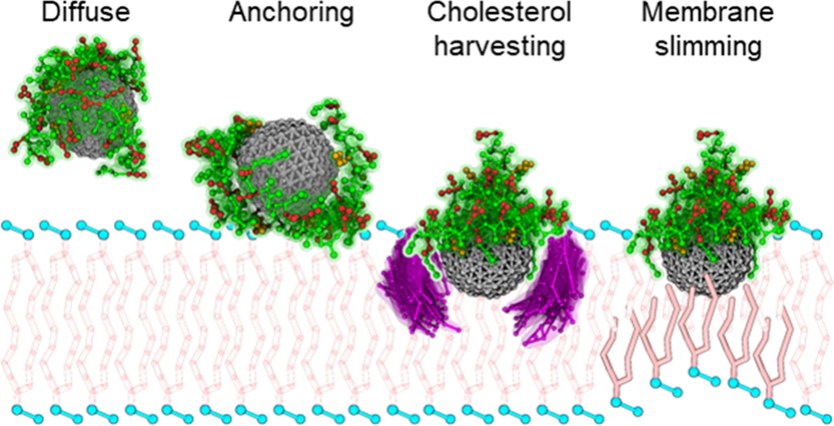

Functionalized metal nanoparticles (NPs) hold great promise as innovative tools in nanomedicine. However, one of the main challenges is how to optimize their association with the cell membrane, which is critical for their effective delivery. Recent findings show high cellular uptake rates for NPs coated with the polycationic cell-penetrating peptide gH625-644 (gH), although the underlying internalization mechanism is poorly understood. Here, we use extended coarse-grained simulations and free energy calculations to study systems that simultaneously include metal NPs, peptides, lipids, and sterols. In particular, we investigate the first encounter between multicomponent model membranes and 2.5 nm metal NPs coated with gH (gHNPs), based on the evidence from scanning transmission electron microscopy. By comparing multiple membrane and (membranotropic) NP models, we found that gHNP internalization occurs by forming an intermediate state characterized by specific stabilizing interactions formed by peptide-coated nanoparticles with multicomponent model membranes. This association mechanism is mainly characterized by interactions of gH with the extracellular solvent and the polar membrane surface. At the same time, the NP core interacts with the transmembrane (cholesterol-rich) fatty phase.

## Introduction

Functionalized metal nanoparticles (NPs) are gaining attention because they display tunable surface chemistry dictated by coating a monolayer of organic molecules (i.e., ligands).^1−5^ These NPs hold promise in numerous biomedical applications from imaging^6^ to cancer therapy,^7^ most of which require the accumulation of NPs inside targeted cells.^8^ Functionalized NPs are typically internalized through energy-dependent pathways like receptor-mediated endocytosis, yet, in some rare cases, they can enter cells through passive diffusion or passive endocytosis.^9,10^ Importantly, all of the above mechanisms are initiated by the nanomaterial’s association with cell membranes.^11,12^ Nonetheless, the exact association mechanism of a NP’s structure with biological barriers continues to be poorly understood.

The NP–membrane interactions are susceptible to the NP’s physicochemical properties, including its size,^13,14^ shape,^15,16^ hydrophobicity,^17^ and ligand density.^18^ Indeed, variations in the monolayer coating can lead NPs to adsorb,^19^ embed,^20^ wrap,^21^ or dangerously rupture cell membranes.^22^ The insights gained so far on the nano-biointerface have boosted the design of membrane-affine monolayers composed of zwitterionic,^23^ PEGylated,^24^ lipid-based,^25^ and jettisoning guest–host ligands.^26^ Other critical factors for modulating cell internalization include environmental variables such as culture media,^27−29^ membrane curvature,^30^ and, of relevance to this study, cholesterol abundance in the membrane.^31^

Among these coating strategies and factors, peptide-based monolayers are effective alternatives that can enhance the internalization rate of NPs while fostering a safe toxicological profile.^32^ In particular, cell-penetrating peptides (CPPs, a family of oligopeptides less than 30 amino acids long) promptly fuse with plasma membranes to eventually deliver cargo into cells, as is often found with viruses.^33,34^ One example is the glycoprotein H (gH) of the herpes simplex virus type 1 (HSV-1), where a specific CPP membranotropic subsequence (aa 625–644) can fuse and translocate into cells without membrane disruption.^35,36^

The transfection mechanisms of viruses have inspired the functionalization of macromolecules with gH625-644 (gH) peptides, which has become an efficient strategy for increasing the internalization rates of NPs. By conjugating gH peptides onto nanomaterials like liposomes,^37^ dendrimers,^38^ brush copolymers,^39,40^ and quantum dots,^41,42^ one can increase the carriers’ fusogenic activity relative to their naked counterparts. This was recently demonstrated by Guarnieri et al. with platinum NPs coated with gH peptides that can translocate cell membranes through endocytosis and passive mechanisms in a size-dependent manner.^43^ Despite the consistently positive results of these peptidic monolayers, the mechanism under which gH extends its membranotropic properties to macromolecular assemblies remains unclear. A molecular understanding of the key NP–membrane interactions that initiate cellular uptake would undoubtedly help optimize peptide-coated metal NPs.

Computational methods are becoming increasingly valuable for investigating the nano-biointerface.^44−46^ Molecular dynamics (MD) simulations are instrumental as they allow studying a controlled set of particles on a molecular scale for time intervals in the order of nanoseconds.^47^ Coarse-graining (CG) is another simulation approach that reduces the phase space’s dimensionality by grouping multiple atoms into individual beads, offering reliable representations of systems otherwise too complex to simulate.^48^ Indeed, CG MD has provided valuable insights into nanomaterial–membrane interactions.^49,50^ However, while there are multiple tools available for parametrizing individual macromolecules,^51,52^ they are rarely combined into model systems that simultaneously contain metal NPs, proteins, lipids, and sterols.

Here, we use CG MD simulations and potential of mean force calculations to investigate the mechanistic pathway under which gH peptides extend their membranotropic properties to metal NPs. For this, we model peptide-coated metal NPs interacting with cholesterol-loaded lipid membranes, supported on the evidence from scanning transmission electron microscopy. Based on our models and results, we propose an association mechanism of peptide-coated NPs with multicomponent model membranes. The gH-coated NPs form a biphasic binding mode halfway embedded into the membranes, simultaneously exploiting the hydrophobic metal core and the amphiphilic peptides.

## Results

### Functionalized gH-Coated NPs Adopt a Biphasic Binding Mode at Lipid Membranes

**Figure 1:**
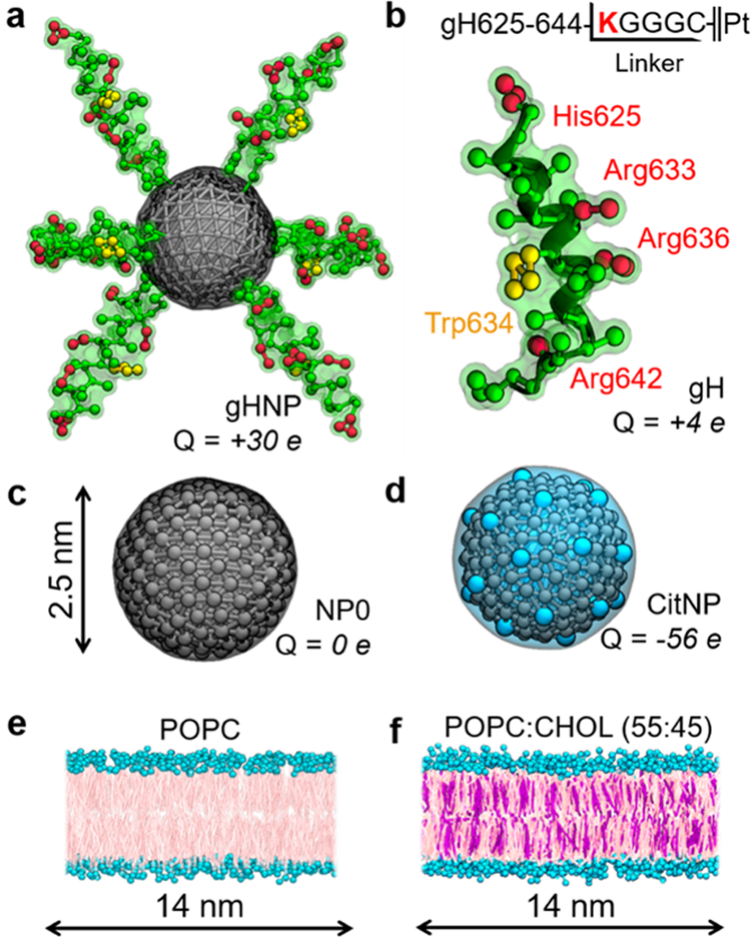
Coarse-grained representation of the studied nanocarriers and lipid bilayers. (a) Spherical metal NP functionalized with six gH peptides. (b) gH peptide and the amino acid linker sequence used for its conjugation to the metal core. (c) Bare hydrophobic NP with a diameter of 2.5 nm. (d) Citrate-capped NP with citrate represented implicitly by −2*e* charges placed in beads uniformly distributed on the NP’s surface.^53^ (e) Pure POPC bilayer. (f) CHOL-loaded POPC bilayer. Neutral amino acids are displayed in green, cationic residues in red, Trp634 in orange, metal beads in gray, citrate beads in cyan, PC headgroups in aquamarine, lipid tails in pink, and CHOL in purple.

We first simulated 2.5 nm functionalized metal NPs interacting with multicomponent model membranes. For this, we built specific coarse-grained (CG) models for each of our studied systems (i.e., four nanocarriers and two membranes), and we parametrized them with the Martini force field. Our four representative nanocarriers were: (i) a metal NP coated with six gH625-644 (gH) peptides (gHNP, Figure 1a), (ii) an individual gH peptide (gH, Figure 1b), (iii) a spherical, purely hydrophobic NP (NP0, Figure 1c), and (iv) a spherical citrate-capped NP (CitNP, Figure 1d). The NP models were built and parametrized with a procedure that has previously reproduced colloidal properties of metal NP suspensions (see the Methods section for details).^53^ All of the carriers were allowed to interact with a pure POPC bilayer (Figure 1e) or with a POPC bilayer loaded with cholesterol (CHOL, Figure 1f). The second membrane used a POPC:CHOL ratio of 55:45 to reproduce the conditions of previous gH fusion experiments.^54^

We examined the association for each carrier–membrane pair using three replicas of 1 μs long coarse-grained (CG) molecular dynamics (MD) simulations. These simulations allowed us to characterize the structural features and the mechanistic pathways followed by different nanocarriers in the presence of our model bilayers. Moreover, we performed multiple replicas of the potential of mean force (PMF) calculations (seven for gHNPs, five for gH, and one for the spherical NP0 and CitNP). Our PMF calculations at a CG resolution qualitatively describe the energy landscape during carrier–membrane association at various incidence angles.

**Figure 2:**
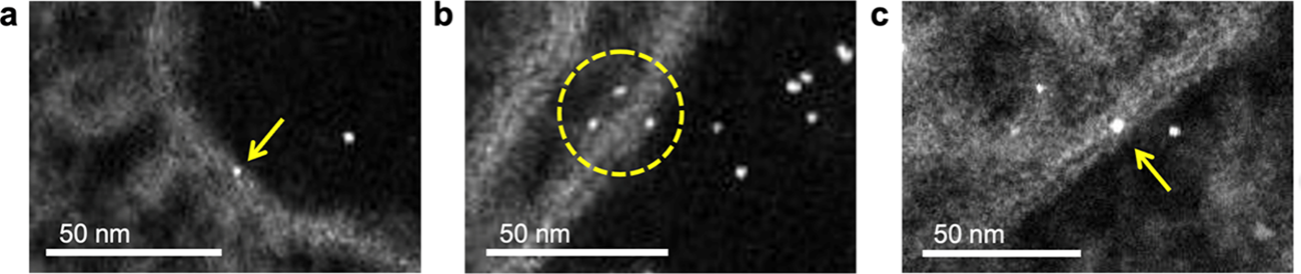
STEM images of 2.5 nm platinum gHNPs interacting with the membrane of HeLa cells after 24 h of incubation at 50 μg mL^–1^ particle concentration. The yellow arrows and dashed circle show single gHNPs at different stages of their translocation (a, b) across cell membranes and (c) endolysosomal membranes.

Our simulations and model systems have been specifically designed to investigate the association mechanism of peptide-coated NPs with the membrane. Such association, in fact, has been demonstrated to occur for these specific metal NPs.^43^ Here, we further confirm these association events by cellular assays and electron microscopy experiments of 2.5 nm platinum NPs coated with the gH oligopeptide. In this case, platinum gHNPs were synthesized, as previously described (diameter 2.6 ± 0.4 nm, Figure S1),^43^ and incubated with human cervix epithelioid carcinoma (HeLa) cells for 24 h (see the Methods section for details). The rested cell culture was then imaged at various regions by scanning transmission electron microscopy (STEM, Figure 2). Extracted STEM images showed single functionalized gHNPs bound to lipid bilayers during different stages of their translocation across cellular (Figure 2a,b) and endolysosomal membranes (Figure 2c).

**Figure 3:**
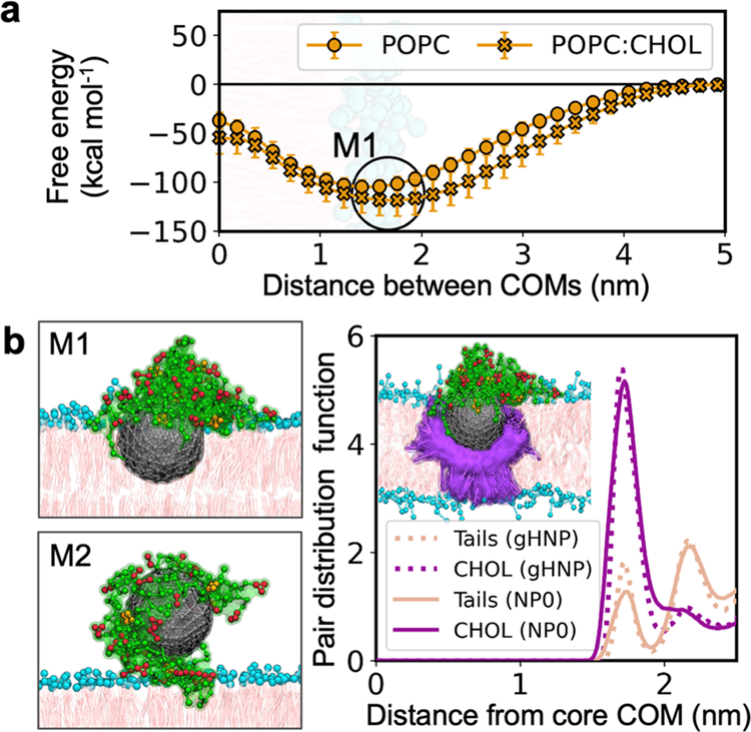
Interaction pattern between gH-functionalized nanoparticles and lipid bilayers. (a) Free energy profiles for the peptide-functionalized gHNPs binding to the POPC and POPC:CHOL membranes. The binding process is defined in terms of the distance between the center-of-mass (COM) of gHNP and the bilayer. (b) Radial distribution function of lipid tails and CHOL as a function of the distance from the core’s COM. The inset superposes multiple frames to illustrate the formation of the CHOL cocoon. The color scheme is the same as that used in Figure 1.

Based on such experimental evidence (Figure 2),^43^ we first used our specific models to study the change in free energy for gHNPs binding to one of the two considered lipid bilayers, i.e., with and without cholesterol. Within the limitations of our approach, the potential of mean force for gHNP-membrane binding was computed along the direction connecting the center-of-mass (COM) of the nanocarrier and the bilayer. Our semiquantitative PMF calculations displayed a free energy basin, labeled M1, at ca. 1.6 nm, regardless of the presence of CHOL (Figure 3a). In this global minimum, the metal core was embedded halfway into the bilayer, forming favorable dispersive interactions with the lipids’ tails. In contrast to the metallic core, the coating peptides remained on the surface of the membrane, establishing a salt-bridge network with the zwitterionic phosphatidylcholine lipid headgroups. The structure of this gHNP-membrane complex is consistent with observations of anionic and mixed-monolayer NPs studied by others.^55−57^

This energy well corresponded to a free energy of association of −104.8 ± 7.1 kcal mol^–1^ at 1.46 ± 0.05 nm for POPC and −118.2 ± 16.5 kcal mol^–1^ at 1.70 ± 0.09 nm for POPC:CHOL. Notably, all our equilibrium CG MD simulations also visited M1. For POPC, the state M1 was reached at 550, 862, and 895 ns. For POPC:CHOL, M1 was reached at 227, 533, and 864 ns (Figure S2). Importantly, once in M1, gHNP always remained stably bound to the membrane until the end of the simulations. For POPC:CHOL, the metallic core is packed more tightly with CHOL molecules (Figure 3b). The radial distribution functions (RDF) computed for CHOL and lipid tails (Figure 3b) demonstrated an increased occurrence of CHOL molecules around gHNP. Moreover, gHNPs also interacted with lipid bilayers exploiting the outer aqueous phase, leading to a biphasic fatty/aqueous binding mode that mined the membranotropic capacities of gH and the hydrophobic character of bulk metals.

Notably, one of our plain CG MD simulation of gHNP with POPC visited a metastable state denoted as M2 (Figure 3b). This ancillary state displays a peptidic cushion formed by gHNP between the membrane and the metallic core. In this simulation, gHNP freely diffused from the bulk solvent into M2 at 126 ns. After ca. 700 ns, gHNP spontaneously fell into the M1 state at 864 ns, confirming the transitory occupancy of the M2 metastable state. Also in this case, gHNP stayed in M1 for the remainder of the simulation.

### Membrane Binding of Primitive gH Suggests a Distinctive Binding Mechanism for gHNPs

**Figure 4:**
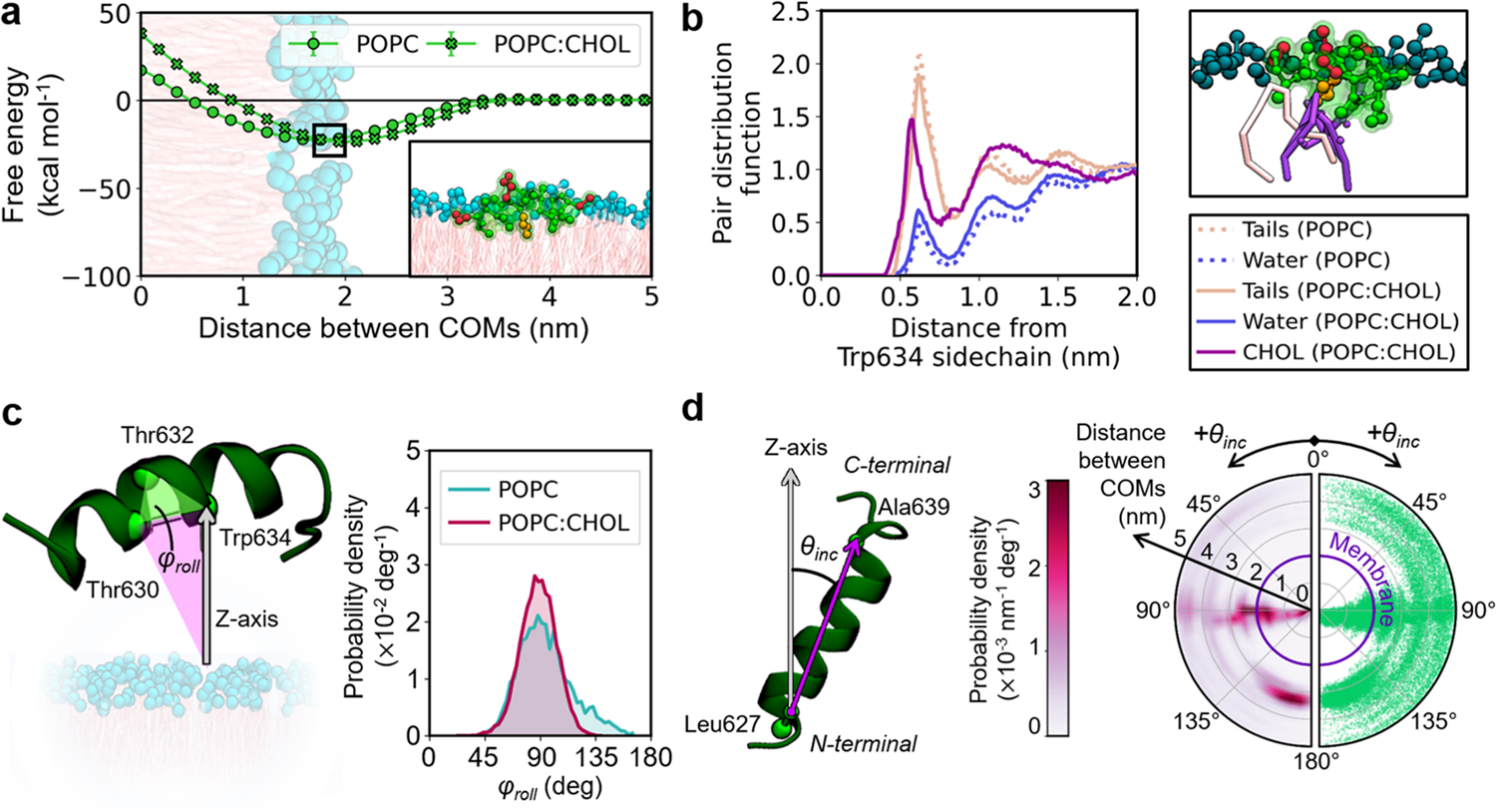
Interaction pattern between gH peptides and lipid bilayers. (a) Free energy profiles for gH binding onto POPC and POPC:CHOL membranes. The binding process is described as the distance between the center-of-mass (COM) of the peptide and the bilayer. The inset shows the bound complex. (b) Radial distribution function of lipid tails, water, and CHOL for Trp634 when bound to POPC and POPC:CHOL (left panel). The gH peptide interacting with a POPC lipid and a CHOL molecule when bound to the membrane (top right panel). (c) Definition of the rolling angle φ_roll_ that describes the rotation of the peptide’s helix around its axis. The angle φ_roll_ is defined in terms of the backbone beads of Thr630, Thr632, and Trp634, shown as green spheres (left panel). The distributions of φ_roll_ are shown for the bound complex found with POPC and POPC:CHOL (right panel). (d) Definition of the polar angle θ_inc_, which describes the peptide’s preferred orientation at varying distances from the membrane. The angle θ_inc_ is defined in terms of the backbone beads of Leu627 and Ala639, shown as green spheres (left panel). The states sampled during the simulations are represented as scattered green points, and the associated probability density is shown as a pink map (right panel). The color scheme is the same as that used in Figure 1.

To understand the membranotropic properties of gHNPs, we compared these NPs with an individual gH peptide interacting with our POPC and POPC:CHOL membranes. For POPC, all five PMF replicas of an individual gH peptide identified the same free energy minimum, in which gH was fused to the membrane (Figure 4a). This optimal state was characterized by a distance between COMs of 1.73 nm (standard deviation <0.01 nm between the five PMF profiles) and a free energy of association to the membrane of −22.3 ± 0.7 kcal mol^–1^. Consistently, our equilibrium CG MD simulations showed the gH peptide, initially placed 3 nm above the bilayer, binding spontaneously to the POPC bilayer 45, 313, and 728 ns after the simulations had started (Figure S3). The peptide always remained stably bound until the end of the simulations. Importantly, these results are consistent with previous circular dichroism and fluorescence quenching experiments, where gH was found to stably adsorb on the surface of phosphatidylcholine (PC) lipid bilayers.^54^

The gH peptide produced similar results in the presence of CHOL. The corresponding PMF profiles again indicated a single minimum, this time at a separation of 1.99 nm (standard deviation <0.01 nm). In this case, the free energy of association with POPC:CHOL was −23.5 ± 0.6 kcal mol^–1^, suggesting a minimal effect of CHOL on gH binding (Figure 4a). Notably, spectrofluorimetric titrations had previously estimated a 10-fold increase in the partition coefficient of gH on POPC:CHOL (55:45) compared to pure POPC.^58^ This difference corresponds to a decrease in free energy of roughly 1.4 kcal mol^–1^, which falls within the statistical error of PMF methods. In light of this, our free energy calculations do hint toward the same experimental trend. Notably, our plain CG MD simulations also showed the peptide’s irreversible binding to the membrane within the same nanosecond timescale as before (38, 46, and 50 ns).

The effect of cholesterol on gH binding was further investigated by characterizing the gH-membrane complex for POPC and POPC:CHOL, as found in our plain CG MD simulations. In particular, we resolved the local chemical environment for gH by computing the RDF of the hydrophobic tails, water, and CHOL for Trp634 (Figure 4b), a residue labeled in fluorescence experiments.^36^ Interestingly, the local density of water and lipids around Trp634 remained nearly constant for both studied membranes. However, the first peak for CHOL displayed a shift toward shorter distances, indicating that Trp634 packed more tightly with CHOL than with the other two components, i.e., lipid tails and water.

The close packaging of CHOL around gH also influenced the rotation of gH along its axis when bound to POPC:CHOL. To illustrate this effect, we monitored the evolution of φ_roll_ (Figure 4c), an angle that described the rolling of the α-helix on the surface of the membrane. In particular, when φ_roll_ ∼90°, the side chain of Trp634 pointed toward the bilayer core, promoting that residue’s insertion. The distribution of the rolling angle φ_roll_ changed from 94 ± 22° in POPC to 88 ± 14° in POPC:CHOL. As expected, these distributions overlap. But CHOL led to a narrower distribution of φ_roll_, suggesting that the gH-CHOL interaction may hamper the rolling of gH peptides on the membrane, stiffening the gH-membrane complex. Notably, for gHNPs bound to POPC and POPC:CHOL, the distribution of φ_roll_ preferred by gH peptides vanished, with each of the peptides adopting a different conformation (Figure S4).

Our PMF calculations also showed that two highly populated peptide conformations were conserved between the POPC and POPC:CHOL systems, as identified by tracking the orientation of the peptide’s α-helix (θ_inc_ in Figure 4d) during peptide binding. The first conformation, found at the bilayer surface, matched the free energy minimum discussed above, in which the peptide sits parallel to the membrane (θ_inc_ ∼90°). Conversely, the second conformation appeared at distances of between 3 and 4 nm from the bilayer midplane. At this stage, the peptide adopted an antiparallel conformation (θ_inc_ ∼180°), meaning that the C-terminal of gH preferentially pointed toward the membrane. This suggests that the charged Arg642 likely initiated the peptide’s anchoring to the phospholipids’ headgroups. Notably, previous mutagenesis experiments have shown that replacing Arg642 (the only arginine residue close to the C-terminal) with a serine inhibits the fusion activity of the peptide,^36,58^ which is consistent with the proposed orientation-specific binding mechanism of gH to the membrane.

Importantly, these results highlight that gH interacts differently with the membrane when alone or attached to the NP. Indeed, we note that the monolayer of gHNPs was grafted onto the core through the C-terminal of gH peptides. In this way, the key interaction of Arg642 for anchoring gH to the membrane (described above) cannot occur in gHNPs. That is, for gHNPs, Arg642 cannot be positioned in such a way as to initiate membrane binding. This key structural difference implies that gHNPs interact and bind to membranes through a different mechanism than gH peptides, as in our simulations. Despite this different binding mechanism for peptide–membrane anchoring, we found that the final bound state of gH and gHNPs ends with the peptide(s) similarly adsorbed on the surface of the membrane. This reflects that despite a distinctive mechanism for membrane adhesion, gHNPs conserve the membranotropic properties of their cell-penetrating peptide component.

### gHNPs Harvest Cholesterol Molecules and Lipid Tails

**Figure 5:**
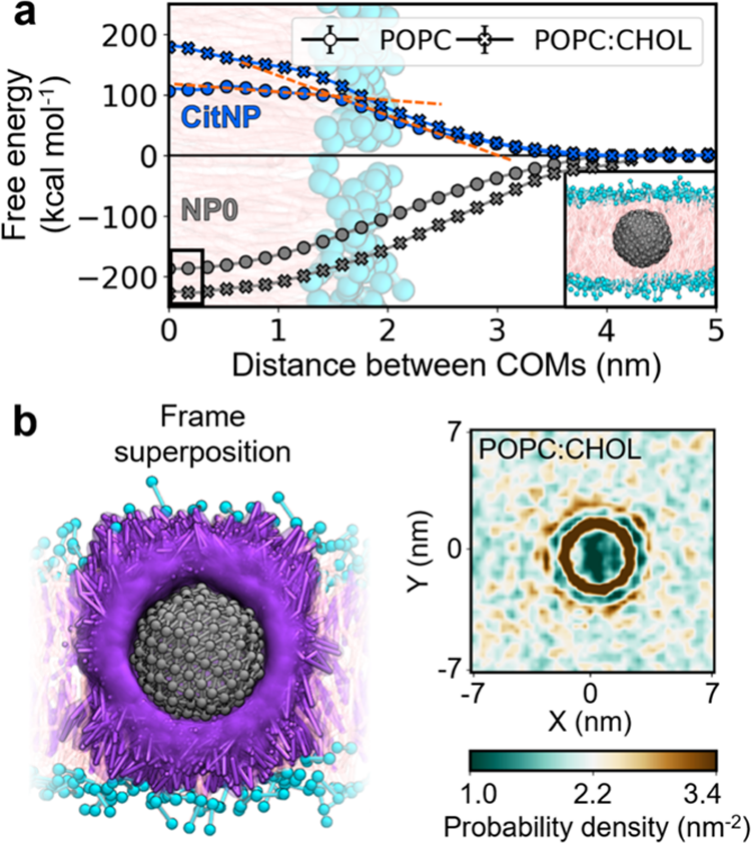
Interaction pattern between naked and capped nanoparticles with lipid bilayers. (a) Free energy profiles for the bare (NP0) and citrate-capped (CitNP) nanoparticles binding to the POPC and POPC:CHOL membranes. The binding process is described as the distance between the center-of-mass (COM) of the NPs and the bilayer. The intersection of the orange dashed lines marks an inflection point of the free energy curve. (b) Density of CHOL after NP0 is spontaneously embedded into POPC:CHOL. CHOL molecules aggregate around the metallic core forming a caging cocoon (left panel). The color scheme is the same as that used in Figure 1.

We proceeded to study the role of the inner metallic core in the overall association of gHNPs with lipid membranes. As for the previous systems, we computed the PMF along the carrier–membrane distance. The free energy profiles displayed a single minimum when a naked NP (NP0) was fully embedded in the bilayer, that is, at 0.0 nm (Figures 5a and S5). The free energy of insertion was −187.1 ± 0.1 kcal mol^–1^ for POPC and −228.1 ± 0.1 kcal mol^–1^ for POPC:CHOL.^59,60^ The accentuation of free energy in CHOL-loaded membranes resulted from a stronger interaction between NP0 and CHOL over NP0 and POPC lipids (Figure 3b).

Our plain CG MD simulations endorsed the results of our PMF calculations on NP0, as we observed the spontaneous insertion of NP0 into both POPC and POPC:CHOL (Figure S5). Moreover, our simulations of NP0 with POPC:CHOL provided deeper insights into the formation of the CHOL cocoon, which we originally observed around gHNP (Figure 3b). We computed the RDF of CHOL around NP0 (Figure 3b), as well as the lateral number density of CHOL in the membrane (Figure 5b). These two metrics showed an oscillatory behavior in the packing of CHOL resulting from the steric hindrance between the CHOL molecules of the first and second solvation shells.

In addition to the purely hydrophobic NP0, we investigated membrane association in the presence of citrate ligands (CitNP), a coating that enables the colloidal stability of metal nanoparticles in polar environments.^53^ In contrast to NP0, the PMF profiles showed a free energy barrier as CitNP approached the membrane, obstructing its translocation across lipid bilayers (Figures 5a and S6). The height of this barrier was 106.5 ± 0.1 kcal mol^–1^ for POPC and 179.3 ± 0.1 kcal mol^–1^ for POPC:CHOL. Both the POPC and POPC:CHOL profiles displayed two different slopes before and after 1.8 nm (Figure 5a), which coincided with the distance at which CitNPs were embedded halfway into the bilayer. This change in the slope illustrated the two forces governing CitNP-membrane fusion, that is, (i) the electrostatic pairing of citrate with the lipid headgroups and the solvent and (ii) the hydrophobic matching of the metallic core with the lipid tails.

The free energy barrier found for CitNP suggests reduced uptake rates of CitNPs compared to gHNPs. Indeed, inductively coupled plasma atomic emission spectroscopy (ICP/AES) experiments previously reported that the uptake of CitNPs by HeLa cells was 12-fold lower than that for gHNPs.^58^ It is important to note that the PMF profiles of CitNP were calculated for a single NP approaching the bilayer. This profile suggests that a CitNP should not be able to be adsorbed or embedded, in its fully charged state, into a lipid bilayer. However, the internalization of these NPs could still take place through alternative mechanisms like receptor-mediated endocytosis or cooperative pathways.^59,60^ Nonetheless, the comparison between CitNPs and gHNPs illustrates how the grafting distribution and flexibility of charged ligands can have opposite effects in the affinity of NPs for lipid bilayers.^58^

In our simulations, as in experiments,^43^ gHNP formed a stable complex with the lipid bilayer without affecting the overall structural integrity of the membrane, unlike other cationic metal NPs that have consistently led to heavy membrane disruption.^61,62^ However, the binding of gHNP caused a local decrease in the area per lipid from 0.65 ± 0.01 to 0.44 ± 0.15 nm^2^ in POPC and from 0.44 ± 0.01 to 0.23 ± 0.09 nm^2^ in POPC:CHOL. This finding reflects a local increase in the bilayer’s density, hinting at a local stiffening of the membrane. The area per lipid of the membrane also changed for individual gH peptides, decreasing to 0.55 ± 0.09 nm^2^ in POPC and to 0.34 ± 0.03 nm^2^ in POPC:CHOL. As for gHNP, the condensation of lipids surrounding gH could be associated with the extracellular release of small molecules, like fluorescent dyes, as observed for pure gH peptides in leakage experiments.^54,58,63^ Our simulations suggest that, either free or grafted, gH peptides preferentially bind to lipid bilayers in a horizontal conformation, pulling neighboring lipids, yet retaining the overall structural integrity of the membrane. These results collectively recover the membranotropic character that gH features during the transfection of HSV-1^54^ and extended to functional platinum gHNPs.

**Figure 6:**
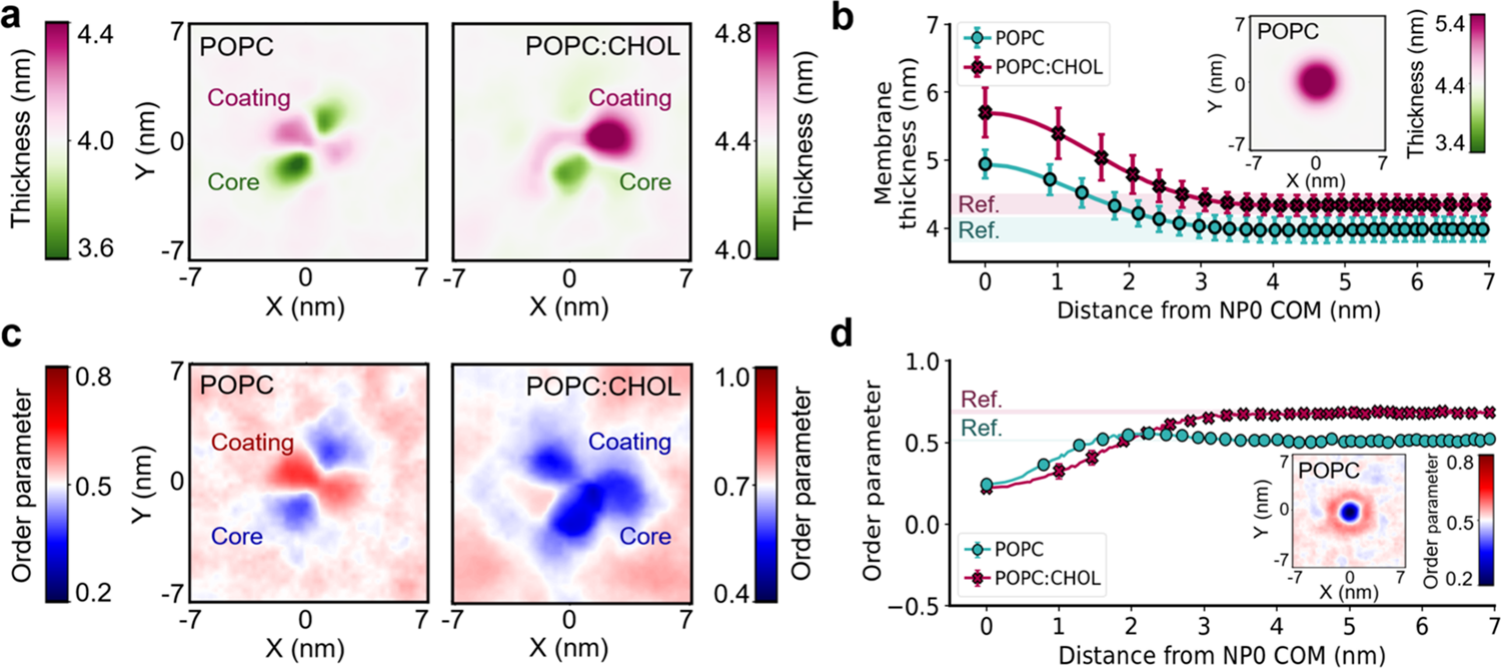
Membrane distortion upon binding of gHNPs and NP0 onto lipid membranes. (a) Top view of the mapped membrane thickness averaged over the frames where gHNP was bound to POPC (left panel) and POPC:CHOL (right panel). The thinner patches match the regions where the core was located, and the thicker areas coincide with the binding spot of the coating peptides. (b) Membrane thickness as a function of distance from the NP0’s center-of-mass (COM). The reference values for the POPC and POPC:CHOL membranes were obtained from separate simulations of equilibrated membranes. The inset shows the membrane thickness of POPC when NP0 was embedded. (c) Top view of the mapped lipid order parameter averaged over time for POPC (left panel) and POPC:CHOL (right panel). (d) Mean lipid order parameter as a function of distance from NP0’s COM. The inset shows the lipid order parameter of POPC when NP0 was embedded.

Beyond a decreased area per lipid, the binding of gHNP led to nonadditive local distortions in the membrane that differed from what was observed for gH and NP0, separately. With gHNP, the membrane thickness increased underneath the coating peptides, leading to values of 4.22 ± 0.18 nm in POPC (6% increase with respect to the 3.97 ± 0.18 nm of an unperturbed membrane) and 4.88 ± 0.18 nm in POPC:CHOL (12% increase with respect to the 4.33 ± 0.15 nm of an unperturbed membrane). In contrast, the binding of individual gH peptides did not change the bilayer thickness of the membranes. Furthermore, for gHNPs, the membranes became thinner at the metallic core’s location, reaching values of 3.55 ± 0.21 nm (11% decrease) in POPC and 4.05 ± 0.16 nm (7% decrease) in POPC:CHOL (Figure 6a). The thinning around gHNP’s metallic core contrasts with the same measurements for NP0. When NP0 was embedded into the membrane, the thickness increased to 4.58 ± 0.19 nm in POPC (15% increase for the nominal value) and 5.40 ± 0.19 nm in POPC:CHOL (25% increase for the nominal value) (Figure 6b). These opposing trends for the spherical core in gHNP and NP0 suggest that the grafted gH plays a key role in modulating the membrane thickness (see the Discussion section).

Similar nonadditive local distortions were also found when assessing the organization of the lipid tails. The binding of gHNP induced a heterogeneous packing of the neighboring lipids. The second-order parameter of the lipid tails increased around the coating peptides in the absence of CHOL (Figure 6c). However, in the presence of CHOL, this effect is overshadowed by the metallic core, which consistently reduces the lipids’ order. In gHNP and NP0, the metallic body acts as an excluded volume that causes the local liquefaction of the membrane (i.e., less lipid order).^64^ The metallic core forces the lipid tails into circumventing it, i.e., bending toward larger angles for the membrane’s normal (*Z*-) axis, thus decreasing the lipid order parameter (Figure 6d). In the presence of CHOL, the effective volume of the nanoparticle increases due to the formation of the CHOL cocoon, extending the area within which the lipids are disordered. Considering all our results, we note that gHNP induces heterogeneous and local membrane alterations unique to the coated nanoparticle and is affected by the membrane’s content of cholesterol.

## Discussion

In this work, we investigated the membrane association mechanism of 2.5 nm metal NPs coated with the gH(625-644) peptide. To this end, we combined coarse-grained (CG) molecular dynamics (MD) simulations and free energy calculations, on the basis of results and evidence from cellular assays, and scanning transmission electron microscopy (STEM, Figure 2).^43^ We examined the interactions between gH-coated NPs (gHNPs) and lipid membranes in terms of the energetic and structural role of the particles’ primordial components, i.e., individual gH peptides and bare metal nanoparticles NP0.

**Figure 7:**
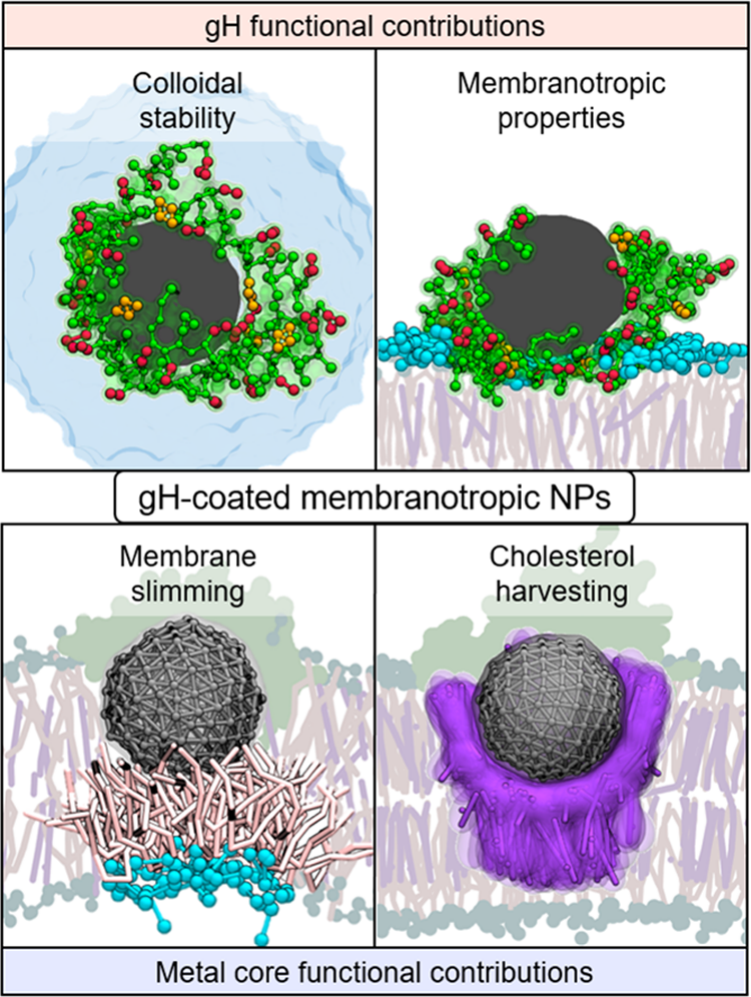
Properties of gH-coated metal NPs. The top panels show the functional contributions of the gH coating, i.e., colloidal stability (left panel) and membranotropic properties (right panel). The gH peptides and lipid headgroups are shown in 3D representations, while the solvent, lipid tails, and CHOL molecules are shown as flat. The bottom panels show the functional contributions of the inner metallic core, i.e., membrane thinning (left panel) and CHOL-harvesting (right panel). The metallic core and its vicinal lipids and CHOL molecules are shown in 3D representations, while the gH peptides and the rest of the membrane are shown as flat. The color scheme is the same as that used in Figure 1.

Our semiquantitative free energy calculations depict a mechanistic landscape for the fusion of individual gH peptides with lipid membranes. We found that the Arg642 residue, which resides at the C-terminal of the gH peptide, is the first residue to interact with the membrane, thus triggering the anchoring of gH to the membrane. This crucial interaction is mainly with the lipid headgroups of the bilayer, leading gH to later rest horizontally on the surface of the membrane (binding free energy −22.3 ± 0.7 kcal mol^–1^ in POPC and −23.5 ± 0.6 kcal mol^–1^ in POPC:CHOL). Notably, this orientation-dependent mechanism agrees with previous mutagenesis and circular dichroism experiments.^36,54,58^ However, in gHNPs, the peptides are grafted onto the metallic core through their C-terminal, thus locating this crucial anchoring Arg642 near the NP core. This structural arrangement blocks Arg642 from interacting with the membrane during binding. Therefore, gHNPs are forced into an alternative binding mechanism. Indeed, our plain CG MD simulations show that the binding of gHNPs onto lipid membranes occurs through nonspecific electrostatic interactions between the polycationic monolayer coating (comprising six gH peptides) and the zwitterionic headgroup of the POPC lipids (Figure 7, top panels). These results support the idea that gH favors membrane binding in gHNPs, even if this occurs through alternative anchoring interactions.

After membrane docking, gHNPs form a stable complex with lipid bilayers. This complex combines structural features from pure gH peptides and naked NP0 nanoparticles (binding free energy −104.8 ± 7.1 kcal mol^–1^ in POPC and −118.2 ± 16.5 kcal mol^–1^ in POPC:CHOL). In the bound complex, the monolayer of gHNPs reorganizes so that the charged amino acids remain on the surface of the membrane, forming a salt-bridge network with the phosphatidylcholine lipid headgroups. At the same time, the inner metallic core is embedded halfway into the lipid bilayer, stabilized by dispersive interactions with the lipid tails (radial distribution function reaches 2.1 in the core’s first solvation shell, Figure 3b). The amphipathicity of gHNPs enables a dual interaction that simultaneously harnesses the high charge density of the cationic coating and the hydrophobicity of the metallic core. Consequently, gHNPs form a biphasic binding mode stabilized by both the outer aqueous phase and the transmembrane fatty phase.

Notably, our simulations and findings are consistent with incubation experiments with HeLa cells and STEM images, which provide further evidence of gHNPs’ biphasic interaction at the membrane. STEM images show some single platinum gHNPs at different stages of translocation across cellular and endolysosomal membranes (Figure 2), as endorsed by previous experiments.^43^ The functionalized gHNPs are persistently found bound to the lipid bilayers, in remarkable consistency with our computational models. In this regard, we account for the complexity of multicomponent mammalian membranes with simulations of a simplified CHOL-containing bilayer. We found that individual gH peptides and bare metal cores (NP0) pack more closely with CHOL molecules than with lipid tails. This mutual preference leads to the formation of a dense CHOL cocoon around the functionalized NP (Figures 3b and 5b). Importantly, higher concentrations of CHOL are known to increase the rigidity of membranes.^65^ Thus, the aggregation of CHOL around gHNP should penalize the bending of the surrounding membrane, as demonstrated in analogous systems (Figure 7, bottom right panel).^66^

Previous MD simulations have studied 3.0 nm anionic NPs embedded (snorkeling) in CHOL-containing membranes without witnessing the CHOL-harvesting effect that we report here.^67,68^ The monolayer coating of our gHNPs was more flexible and less charged than those discussed in the previous studies. Specifically, those simulations used NPs coated with 134 short (3-bead-long) ligands with a charge of −1*e* each (i.e., total charge of −134*e*), while our gHNPs were coated (as determined experimentally) by six amphipathic peptides with a charge of +5*e* each (i.e., a total charge of +30*e*). This suggests that the formation of the CHOL cocoon is affected by the NP charge, ligand grafting density, and ligand flexibility. This result endorses the complex cross-dependence between NP features and their interactions with membranes, and how NP features can be tuned to achieve optimal therapeutic performance.

Experiments have previously determined that platinum gHNPs can be passively internalized by HeLa cells.^43^ In this context, our simulations reveal that the binding of gHNPs causes local distortions on the lipid membrane that may promote the particles’ passive uptake. As a matter of fact, gHNPs decrease the area per lipid of the membranes to 0.44 ± 0.15 nm^2^ in POPC and 0.23 ± 0.09 nm^2^ in POPC:CHOL. Intriguingly, this condensation of the lipids indicates a local increase in the mass density of the bilayer that should penalize the bending of the membrane. While the membrane wrapping rates require further investigation, we note that these would indeed enable small gHNPs to translocate into the cytosol or escape lysosomal compartments passively. Furthermore, when gHNPs are bound to the membrane, the metallic core dangles from the upper (extracellular) leaflet, attracting the lipid tails of the lower (intracellular) leaflet. The hydrophobic pairing between the metal and the lipid tails thins the bilayer thickness by 11% in POPC and 7% in POPC:CHOL, likely facilitating the opening of transmembrane channels at selected patches (Figure 7, bottom left panel). Notably, the observed thinning of the membrane requires that the metallic core is only partially embedded into the membrane, explaining why the passive translocation of bigger platinum gHNPs is reduced in experiments.^43^

Taken together, our extended CG MD simulations suggest that the fusion of gHNPs onto membranes results in a stable biphasic state that is consistent with our STEM imaging experiments (Figure 2).^43^ Our results show distinct yet synergistic effects of the two main components of gHNPs, i.e., the peptide and the NP core. The former component drives membrane fusion, while the latter enhances core embedding into the membrane. The dynamic characterization of the gHNP-bilayer complex revealed that the membrane suffers nonadditive alterations for those observed for free gH peptides and bare NPs.

## Conclusions

We investigated the membrane association of 2.5 nm metal NPs functionalized with the cell-penetrating peptide gH(625-644) using coarse-grained (CG) molecular dynamics (MD) simulations and semiquantitative free energy calculations. Our simulations showed that the functionalized gHNPs conserve the membranotropic properties of the primordial gH peptides, yet their binding process follows a different mechanism. Interestingly, the membranotropic properties of gHNPs arise from nonspecific electrostatic interactions between the polycationic monolayer and the polar lipid headgroups.

Upon binding, gHNPs form a biphasic complex with the membrane, with the coating peptides spread on the polar surface of the membrane and the metallic core embedded halfway into the fatty transmembrane phase. This biphasic binding mode is consistent with our cellular assays and STEM imaging experiments (Figure 2).^43^ We also found a selective thinning of the membrane at the gHNP’s location. This arises from the partly inserted NP core, which attracts the lipid tails of the intracellular leaflet. We hypothesize that these local membrane distortions are a preparatory step for the cellular internalization observed in experiments elsewhere.^43^ Within the limitations of our models and configurational sampling, our results and mechanistic details of NP–membrane association may help explain the alterations that different nanomaterials induce onto the receptive multicomponent membranes.

## Methods

### System Preparation

The initial structure of the glycoprotein H from the herpes simplex virus type I was taken from the Protein Data Bank with the entry code 2LQY.^69^ The peptide was mapped into the Martini v2.2P force field using the *martinize.py* script. An elastic network consisting of a series of harmonic potentials was used to retain the secondary structure of the protein.^70^ The elastic network used a force constant of *k*_b_ = 500 kJ mol^–1^ nm^–2^, and it was applied to all of the protein bead pairs at a distance between 0.5 and 0.9 nm. The peptide was immersed in a box of polarizable (refPOL)^71^ water molecules with a NaCl concentration of 150 mM.^72^ The system was then minimized with the steepest descent method, and simulated for 10 ns with a v-rescale thermostat (310 K, τ_B_ = 2.0 ps) and a Berendsen barostat (1 bar, τ_P_ = 5.0 ps, κ = 4.5 × 10^–4^ bar^–1^).^73^ Then, the system was equilibrated for 1 μs using the Parrinello–Rahman barostat (τ_P_ = 12.0 ps).^74^

The metallic core of the NPs was assembled by uniformly placing 187 beads on a sphere of diameter 2.5 nm, a size chosen to match the corresponding experimental conditions.^43^ The NP beads were assigned the hydrophobic bead type C1, as in previous coarse-grained studies that reproduce the experimental dispersion state of metal colloids.^53,75^ To retain the eccentricity of the sphere, each bead was bonded through a harmonic potential (*k*_b_ = 2250 kJ mol^–1^ nm^–2^) to its six nearest neighbors and its radially opposing neighbor (antipodal bead). The NPs were then minimized with the steepest descent method.^53^ The peptide-coated NPs consisted of a hydrophobic core coated with six peptides, as resolved experimentally, placed at the cardinal points of the sphere.^76^ Notably, gHNPs were functionalized with a modified version of gH, in which a glycine linker is added to the C-terminal to facilitate the NP’s synthesis.^43^ The peptide-bearing NPs were equilibrated following the same procedure as for gH in water (see above).

The citrate-capped metal NPs are studied using an implicit citrate model in which a hydrophobic core is assigned a partial charge to selected surface beads. In this kind of model, the beads representing citrate are covalently bound to the core. It is noteworthy that in contrast to chelation and multipolar interactions, the complexation of citrate onto noble metal surfaces takes place through stiff chemical bonds that dampens ion competition. In our case, we added a net charge of −2*e* to 28 beads (total charge of −56*e*), following a previously reported protocol that reproduced the experimental dispersion state of these colloids.^53^ This specific model was parametrized to reproduce the colloidal stability of citrate-capped gold NPs. Nonetheless, the mesoscopic behavior of bulk gold and platinum (i.e., their hydrophobicity) is comparable and distinguishing between noble metals goes beyond the reach of the Martini force field.

In this study, we prepared two model membranes with the *insane.py* Martini script.^51^ The first of these bilayers consisted of pure POPC, whereas the second contained CHOL at a molar ratio of 55:45 (POPC:CHOL). The force field parameters for CHOL were taken from Daily et al.^77^ The generated structures were parallel to the *XY* plane and had initial dimensions of 14 × 14 nm^2^. For their equilibration, the lipid molecules were fully solvated by leaving a minimum distance of 2 nm between the lipids and the box edges in the *Z*-direction.^78^ The membranes were minimized using the steepest descent method and simulated for 10 ns with a v-rescale thermostat (310 K, τ_B_ = 2.0 ps) and a semi-isotropic Berendsen barostat (1 bar, τ_P_ = 5.0 ps, κ = 4.5 × 10^–4^ bar^–1^).^73^ Then, the system was equilibrated for 1 μs, using the Parrinello–Rahman semi-isotropic barostat (τ_P_ = 12.0 ps).^74^ The convergence of the bilayer thickness, area per lipid, and acyl order parameters during these simulations verified the equilibration of the membranes.

The composition of our model membranes was motivated on previous works by Galdiero et al.^35,79^ and Vitiello et al.^54^ These works compared the fusion of gH peptides onto POPC and POPC:CHOL membranes at the same 55:45 molar ratio. Also, these studies used fluorescence spectroscopy, neutron reflectivity, and electron-spin resonance spectroscopy to demonstrate that gH peptides preferably bind to the CHOL-containing membranes. The trends in binding free energies reported in such studies were used as benchmarking data for CG models.

### MD Simulations and Free Energy Calculations

All of our production simulations involved one of the model membranes (POPC or POPC:CHOL) in the presence of gH or an NP. For this, we extracted the last frame of the equilibration runs of the two respective components, and they were merged into a single simulation box, leaving 5.0 nm between their centers of mass (along the *Z*-axis). The systems were immersed in a box of polarizable (refPOL)^71^ water at a salt concentration of 150 mM^72^ before they were subjected to a minimization with the steepest descent method. The systems were then thermalized and pressurized in a 10 ns long simulation, applying a v-rescale thermostat (310 K, τ_B_ = 2.0 ps), a semi-isotropic Berendsen barostat (1 bar, τ_P_ = 5.0 ps, κ = 4.5 × 10^–4^ bar^–1^),^73^ and a timestep of 10 fs. Then, the production runs were started using the Parrinello–Rahman semi-isotropic barostat (τ_P_ = 12.0 ps)^74^ and increasing the timestep to 20 fs. In all our simulations, the Lennard-Jones and electrostatic interactions were truncated at 1.2 nm. Long-range electrostatics were computed with the fourth-ordered PME method.^80^ Frames were saved every 40 ps for analysis. The Gromacs-v5.1.4 MD engine was used for the entirety of the work.^81^ We performed three 1 μs long replica simulations for each of our systems. Notably, the binding times reported here are indications of the timescale on which binding occurs rather than quantitative measurements.

We adopted a semiquantitative potential of the mean force (PMF) scheme for calculating the free energy of association between carriers (peptides or NPs) and membranes, as implemented in other computational studies involving nanomaterials and membranes.^20,82^ In this case, the selected collective variable (CV) was the *Z*-component of the distance between the center-of-mass of the carriers and the bilayer. The chosen CV for describing the binding process relies on two assumptions. These are the spherical symmetry of the NP and the flatness of the model membrane. The spherical symmetry of our gHNPs was corroborated by their near-zero eccentricity (0.13 ± 0.02).^5^ As for the membranes, we computed the transversal mass density of their phosphate beads (i.e., the density along the *Z*-axis, Figure S7). The density profile showed two narrow peaks at ±[1.96 ± 0.26] and ±[2.15 ± 0.24] nm for POPC and POPC:CHOL, respectively, thus demonstrating planar conformations in average.

To obtain our PMF profiles, we first performed a steered MD simulation to pull both components toward one another and sampled the reaction coordinate. With this method, we applied a harmonic potential (*k*_bias_ = 2000 kJ mol^–1^ nm^–2^) the equilibrium value of which, starting at 5.0 nm, shrank at a rate of 0.15 nm ns^–1^. Then, 51 frames were extracted from the trajectory to sample the CV every 0.1 nm. The free energy calculations relied on the umbrella-sampling technique. With this method, the windows extracted from our steered MD trajectory were used as initial configurations for simulations sampling a limited range of the CV. In these simulations, the original value of the CV at each window was restrained by a harmonic potential (*k*_bias_ = 2000 kJ mol^–1^ nm^–2^). Then, the windows were simulated for 100 ns each, saving the CV every 1 ps and using the same parameters described above for the unbiased runs. The simulated time was 150 μs in total. The semiquantitative free energy profiles were finally reconstructed by merging the CV’s histograms of each simulated window with the weighted histogram analysis method (WHAM).^83^

We performed seven replicas for the PMF profiles of gHNPs, five replica profiles of gH peptides, and one replica for the perfectly spherical NPs, i.e., NP0 and CitNP. The difference between each replica was the starting angle of the nanocarrier with respect to the membrane. The angle of each replica was chosen randomly from a uniform distribution of solid angles.

### Trajectory Analysis

In this study, two geometrical parameters were computed for gH-containing simulations: the polar angle θ_inc_ and rolling angle φ_roll_. The first of these is defined as the angle between the peptide’s α-helix and the vector normal to the bilayer (*Z*-axis). The axis of the helix was determined as the vector from the backbone bead of Leu627 to the backbone bead of Ala639. Angles of θ_inc_ = 0 and 180° indicate that the membrane is closest to the N-terminal and C-terminal, respectively (Figure 1a). In contrast, φ_roll_ is defined as the torsion angle between two planes defined by three vectors. The first vector is the *Z*-axis, the second is the vector from Trp634 to Thr630, and the third is the vector from Thr630 to Thr632. According to the bonded parameters of the peptide (as assigned by the Martini v2.2P force field), at φ_roll_ ∼90°, the side chain of Trp634 points toward the bilayer’s interior.

The radial distribution functions (RDF) of unit A with respect to unit B were calculated using eq 1, where δ_D_(*r*) is Dirac’s Delta function, and *N*_A_ and *N*_B_ are the number of beads in units A and B, respectively.
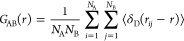
1All of the bidimensional plots presented here were mapped from 50 × 50 grids. The membrane thickness was calculated as the mean distance between the three nearest phosphate beads and each grid point. The lipid order parameter was calculated from Legendre’s second-order polynomial (eq 2), where the ensemble is averaged over time, molecules, and beads. The angle of bead *i*, namely, ω_*i*,*z*_, is the angle formed between the *Z*-axis and the vector uniting bead *i* – 1 with bead *i* + 1. The trajectory analysis was carried out with in-house scripts using the MDAnalysis library from Python.^84^
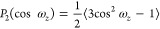
2Lastly, the area per lipid was calculated by performing a Voronoi tessellation with the *X*–*Y* coordinates of selected beads for each frame in the trajectory. For this analysis, we used the phosphate PO4 bead of lipids, the ROH bead of CHOL molecules, and the nanocarriers’ beads lying on the surface of the bilayer. The surface of the bilayer was defined as the space between the highest and the lowest phosphate bead at each frame. The nanocarriers’ beads were only used during the Voronoi tessellation step, yet they were excluded when calculating the averages and deviations herein reported. In this way, our values account for the space occupied by a bound peptide or NP. The reported values are time averages for the upper leaflet (i.e., the leaflet supporting the carrier).^86^

### Cell Culture and Transmission Electron Microscopy (TEM)

Human cervix epithelioid carcinoma (HeLa) cells (ATCC) were cultured in Dulbecco’s modified Eagle’s medium (DMEM, Invitrogen) supplemented with 10% (v/v) fetal bovine serum (FBS, Hyclone), 100 U mL^–1^ penicillin, and 100 mg mL^–1^ streptomycin (Invitrogen). Cells were maintained in an incubator in a humidified controlled atmosphere at 37 °C and 5% CO_2_. For TEM observations, HeLa cells were incubated for 24 h with platinum NPs that were 2.5 nm in diameter and functionalized with gH peptides, as previously reported.^43^ The cells were then processed as described elsewhere.^85^ The images were acquired in STEM mode working in high-angle annular dark-field (HAADF) geometry, using an FEI Tecnai F20 transmission electron microscope operating at 200 kV and equipped with a Schottky field emission gun.

## Supporting Information Available

TEM images of the gHNP colloid synthesized together with the nanoparticles’ size distribution; temporal evolution of the distance between the centers of mass of the model membranes and the nanocarriers (i.e., gH, NP0, CitNP, and gHNP) in the CG MD simulations; probability density of the angle φ_roll_ for the peptides coating the gHNPs when interacting with POPC and POPC:CHOL; and lateral mass density of the phosphate beads in the equilibrated POPC and POPC:CHOL model membranes (PDF)
