# Supplementary material for: Association Mechanism of Peptide-Coated Metal Nanoparticles with Model Membranes: A Coarse-Grained Study
Source: J Chem Theory Comput. 2021 Jun 2;17(7):4512–23. doi: 10.1021/acs.jctc.1c00127 (PMC8280734; doi:10.1021/acs.jctc.1c00127)
Supplement: Supplementary file 1 — ct1c00127_si_001.pdf [file ct1c00127_si_001.pdf]

Supporting information

Association mechanism of peptide-coated metal nanoparticles with model membranes: A coarse-grained study

Sebastian Franco-Ulloa[1,†], Daniela Guarnieri[2], Laura Riccardi[1], Pier Paolo Pompa[3], and Marco De Vivo[*,1]

1. Molecular Modeling and Drug Discovery Lab, Istituto Italiano di Tecnologia, via Morego 30, 16163 Genova, Italy
2. Dipartimento di Chimica e Biologia "A. Zambelli", Università degli Studi di Salerno, Via Giovanni Paolo II 132, l-84084, Fisciano, Salerno, Italy
3. Nanobiointeractions & Nanodiagnostics, Istituto Italiano di Tecnologia, via Morego 30, 16163 Genova, Italy.

† Present address:
Expert Analytics, Møllergata 8, 0179, Oslo, Norway

* Corresponding author:
Dr. Marco De Vivo – Email: marco.devivo@iit.it

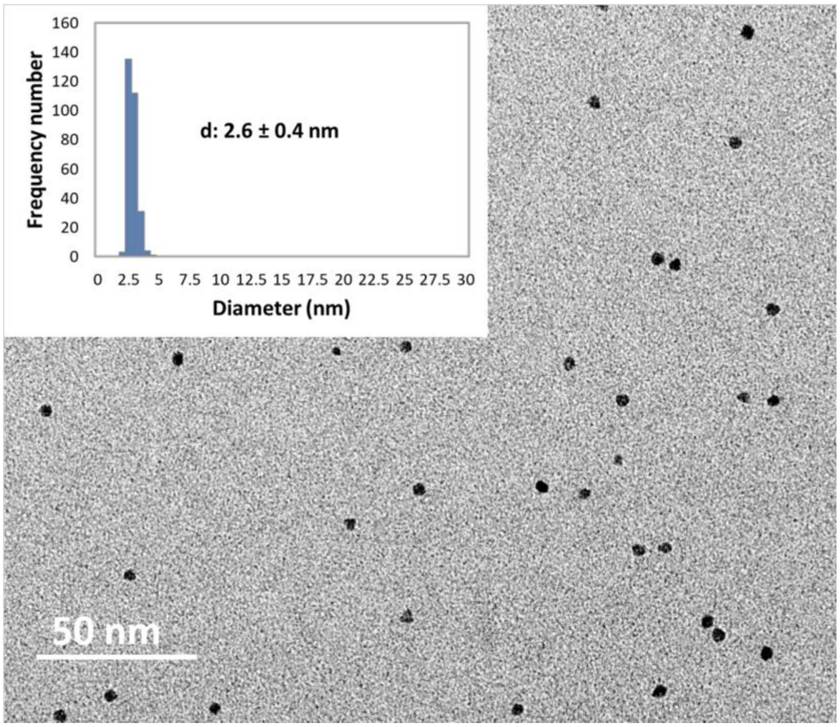

Figure S1. TEM images and size distribution of the synthesized (diameter = 2.6 ± 0.4 nm).

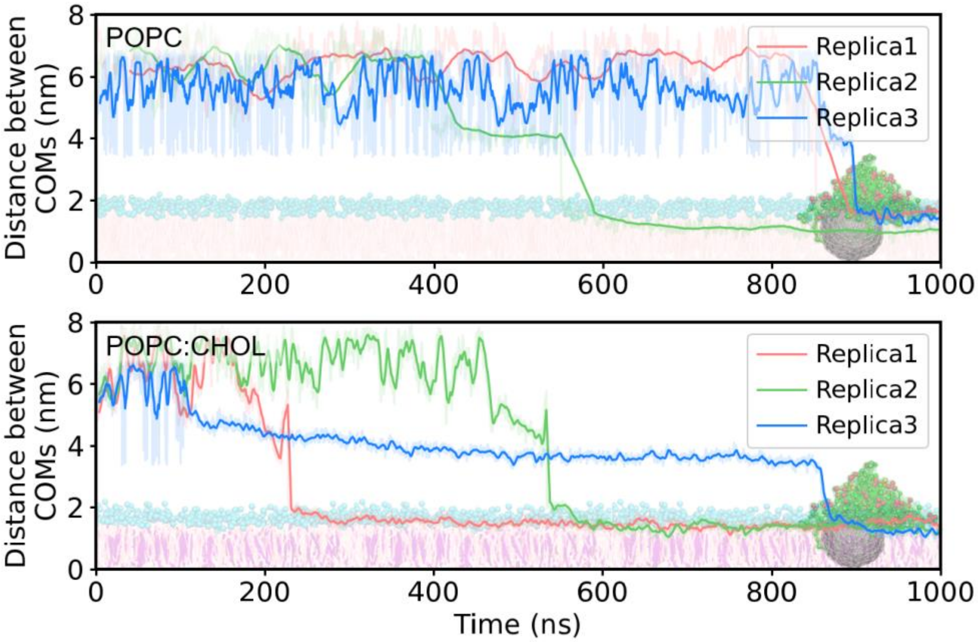

Figure S2. Distance between the COM of the functionalized nanoparticle gHNP and the COM the membrane for each of the three equilibrium CG MD replica simulations. The two membranes employed throughout the study were pure POPC (top) and POPC:CHOL (55:45, bottom).

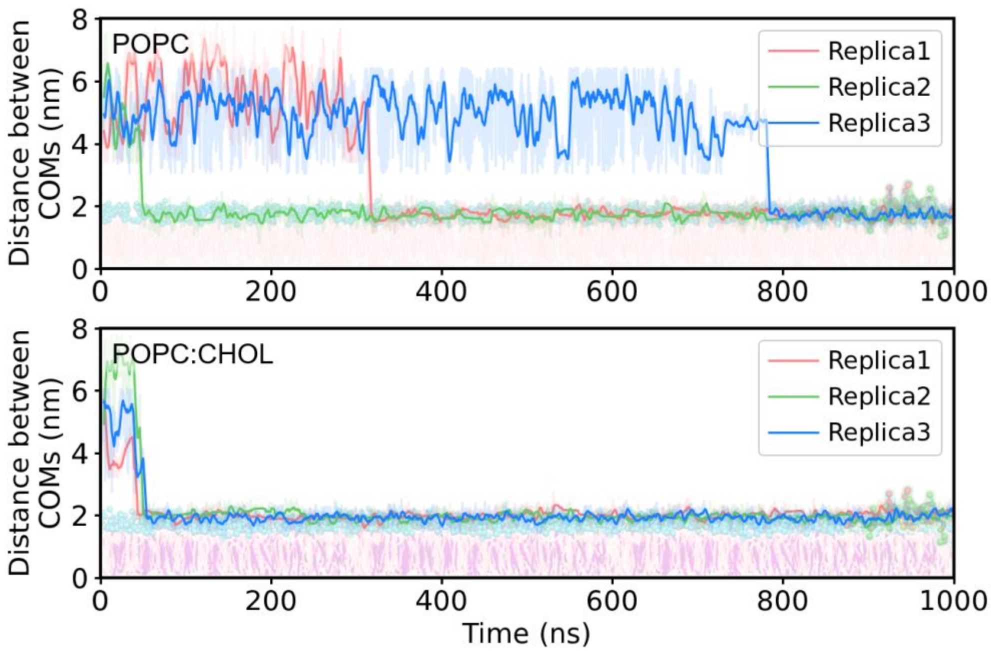

Figure S3. Distance between the COM of the gH peptide and the COM the membrane for each of the three equilibrium CG MD replica simulations. The two membranes employed throughout the study were pure POPC (top) and POPC:CHOL (55:45, bottom).

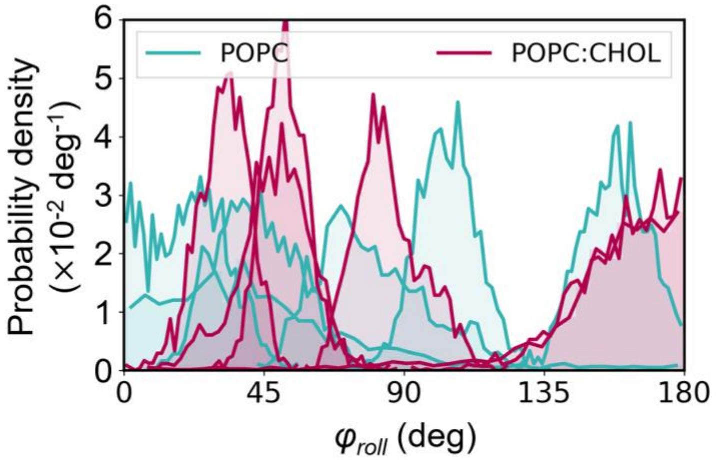

Figure S4. Distribution of the rolling angle φ_{roll} for each of the six peptides grafted to the functionalized nanoparticles gHNP. The distributions are shown for both POPC (green) and POPC:CHOL (red) membranes.

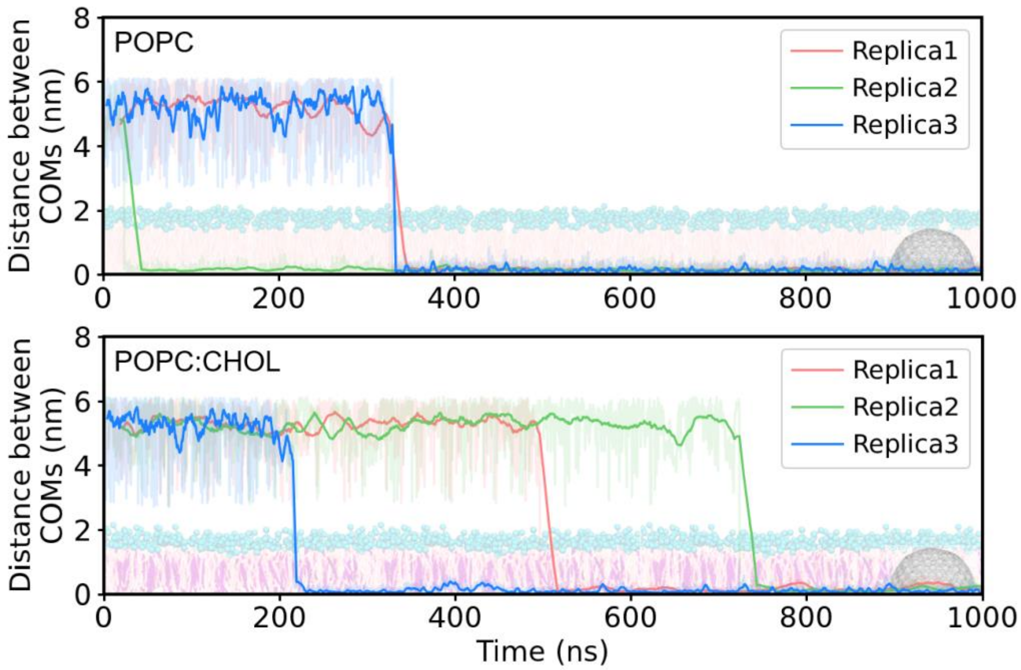

Figure S5. Distance between the COM of the pristine nanoparticle NP0 and the COM the membrane for each of the three equilibrium CG MD replica simulations. The two membranes employed throughout the study were pure POPC (top) and POPC:CHOL (55:45, bottom).

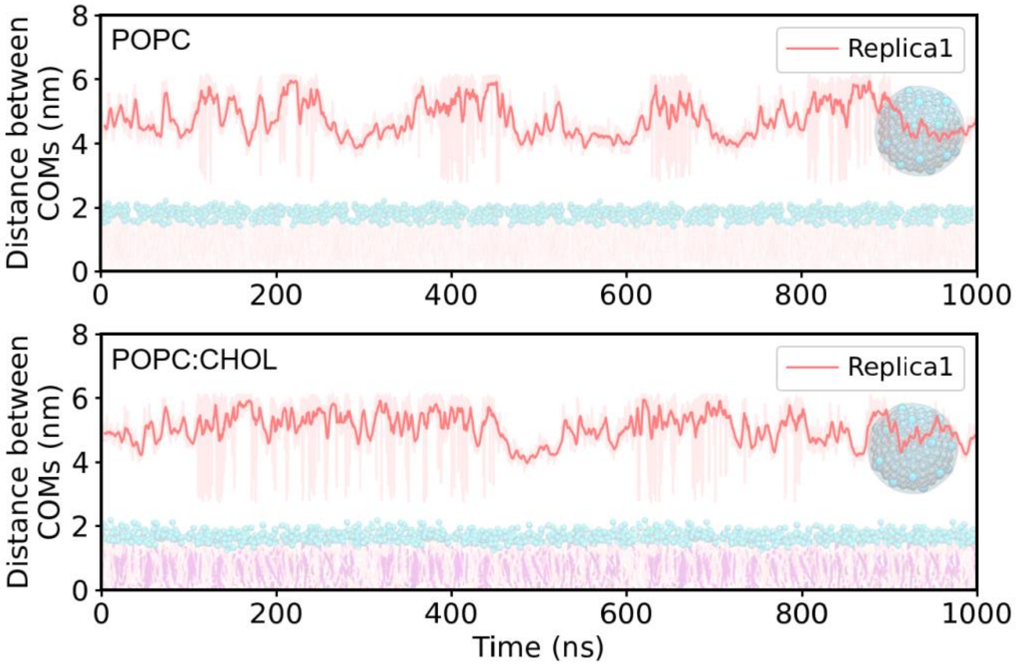

Figure S6. Distance between the COM of the citrate-capped nanoparticle CitNP and the COM the membrane. The two membranes employed throughout the study were pure POPC (top) and POPC:CHOL (55:45, bottom).

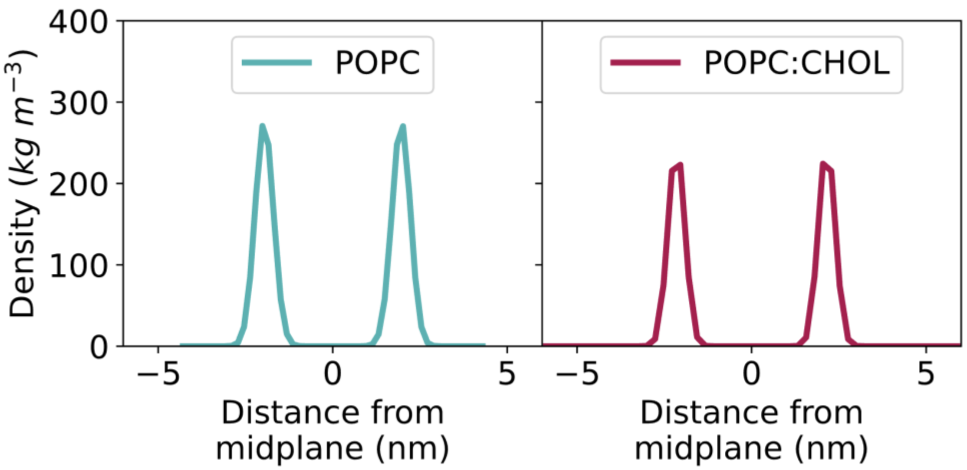

Figure S7. Transversal mass density of the phosphate headgroups (i.e., PO4 beads) for the equilibrated POPC and POPC:CHOL membranes. The peaks of the distributions are located at ± [1.96 ± 0.26] nm and ± [2.15 ± 0.24] nm for POPC and POPC:CHOL respectively.
